# Genetic variation and potential for genetic improvement of cuticle deposition on chicken eggs

**DOI:** 10.1186/s12711-019-0467-5

**Published:** 2019-06-04

**Authors:** Ian C. Dunn, John A. Woolliams, Peter W. Wilson, Wiebke Icken, David Cavero, Anita C. Jones, Fiona Quinlan-Pluck, Gareth O. S. Williams, Victor Olori, Maureen M. Bain

**Affiliations:** 1 The Roslin Institute, University of Edinburgh, Easter Bush Campus, Midlothian, EH25 9RG Scotland UK; 2 Lohmann Tierzucht, 7454 Cuxhaven, Germany; 3 H&N International, 27472 Cuxhaven, Germany; 4 School of Chemistry, University of Edinburgh, Joseph Black Building, Edinburgh, Scotland UK; 5 Aviagen, Midlothian, Scotland UK; 6 College of Medical, Veterinary and Life Sciences (MVLS), IBAHCM, University of Glasgow, Glasgow, Scotland UK

## Abstract

**Background:**

The cuticle is an invisible glycosylated protein layer that covers the outside of the eggshell and forms a barrier to the transmission of microorganisms. Cuticle-specific staining and in situ absorbance measurements have been used to quantify cuticle deposition in several pure breeds of chicken. For brown eggs, a pre-stain and a post-stain absorbance measurement is required to correct for intrinsic absorption by the natural pigment. For white eggs, a post-stain absorbance measurement alone is sufficient to estimate cuticle deposition. The objective of the research was to estimate genetic parameters and provide data to promote adoption of the technique to increase cuticle deposition and reduce vertical transmission of microorganisms.

**Results:**

For all pure breeds examined here, i.e. Rhode Island Red, two White Leghorns, White Rock and a broiler breed, the estimate of heritability for cuticle deposition from a meta-analysis was moderately high (0.38 ± 0.04). In the Rhode Island Red breed, the estimate of the genetic correlation between measurements recorded at early and late times during the egg-laying period was ~ 1. There was no negative genetic correlation between cuticle deposition and production traits. Estimates of the genetic correlation of cuticle deposition with shell color ranged from negative values or 0 in brown-egg layers to positive values in white- or tinted-egg layers. Using the intrinsic fluorescence of tryptophan in the cuticle proteins to quantify the amount of cuticle deposition failed because of complex quenching processes. Tryptophan fluorescence intensity at 330 nm was moderately heritable, but there was no evidence of a non-zero genetic correlation with cuticle deposition. This was complicated furthermore by a negative genetic correlation of fluorescence with color in brown eggs, due to the quenching of tryptophan fluorescence by energy transfer to protoporphyrin pigment. We also confirmed that removal of the cuticle increased reflection of ultraviolet wavelengths from the egg.

**Conclusions:**

These results provide additional evidence for the need to incorporate cuticle deposition into breeding programs of egg- and meat-type birds in order to reduce vertical and horizontal transmission of potentially pathogenic organisms and to help improve biosecurity in poultry.

**Electronic supplementary material:**

The online version of this article (10.1186/s12711-019-0467-5) contains supplementary material, which is available to authorized users.

## Background

The cuticle is a very thin and invisible layer that is deposited on the outside of the egg [1] [for an illustration (see Additional file 1: Figure S1)] and, therefore, there are few studies on this component of the biology of the egg, although it serves an important function. By filling the gas exchange pores, the cuticle prevents bacterial contamination of the egg contents [2–4]. The more pronounced deposition of cuticle on the eggshells of aquatic birds [5, 6], which are faced with significant microbial challenges, is evolutionary evidence that the trait is important for protection of the egg in dirty environments [7] and is genetically controlled. More directly, in a previous study on Rhode Island Red hens, we demonstrated that the size of the genetic component for deposition of cuticle on the eggs was substantial [4]. In that study, we used quantification of a cuticle-specific stain to measure cuticle deposition using reflectance spectroscopy. The staining is related to protein quantity [8], which is linearly related to the depth of the protein layer, as determined by microscopy [9]. More importantly, for a functional application, we observed that within the normal range of variation in cuticle deposition, there was a significant effect on bacterial penetration of eggs [3, 4], with bacteria almost never penetrating eggs that had the most cuticle deposition, whereas those with a poor cuticle were frequently penetrated. We extended these studies to include more breeds of chicken (layers and broilers) and strains of bacteria [3] and obtained results that are consistent with the observation that complete removal of the cuticle increases both water and particle penetration [10, 11].

Vertical and horizontal transmission of bacteria in chickens can threaten embryo development and compromise biosecurity [12, 13], thus improving cuticle deposition on eggs is a potentially worthwhile breeding goal for commercial poultry since it could reduce pathogen entry. Improving the egg’s natural barrier to microbial entry will strengthen and be complementary to the already large physical separation that exists in the poultry industry between offspring and parent from the use of artificial incubation of eggs. This separation is arguably one of the keys to the biosecurity of commercial production.

In our previous work on the Rhode Island Red breed, we estimated the heritability for cuticle deposition at only one time-point during the hen’s productive life [4]. It is also unclear how widely selection for cuticle deposition could be applicable, for example in broiler production. Furthermore, although there is a widespread belief that egg color and cuticle deposition may be associated [14], our previous study found only a small negative genetic correlation between these two traits [4] and failed to show any physiological relationship or dependence between egg color and cuticle deposition [15].

Thus, the aim of the current study was to improve confidence in the measurement of cuticle deposition as a tool for genetic selection, by both replicating and extending our previous findings, by extending the analyses to a White Leghorn (white-egg layer) and a broiler meat-type breed. These breeds have very different genetic backgrounds and can be clearly separated by DNA analyses [16]. In addition, the predictive ability of a measure of cuticle deposition, which is recorded early in the laying period, on future performance was investigated. This knowledge would be valuable to geneticists since (1) qualitatively, it would demonstrate that cuticle deposition is an attribute of a hen’s whole productive lifetime rather than of a short interval within it, and (2) quantitatively, it would reduce the number of measurements that need to be made during a lifetime. Developing a method that requires less specialized equipment or simplifies the measurement of cuticle deposition is also important for incorporating this trait into standard breeding practices. This was explored by comparing single wavelength absorbance with results from a Minolta colorimeter, and by measuring the auto-fluorescence of cuticle proteins under UV excitation, which would avoid the need for staining.

## Methods

### Chicken breeds

We used five breeds of chicken to determine genetic parameters that are relevant to the suitability of the trait for selection.

#### Breed 1

Hens for breed 1 originated from a single generation of the Rhode Island Red (RIR) breed, which is a pure breed laying brown eggs and a contributor to the male that was used to produce the Lohmann Brown commercial layers (Lohmann Tierzucht GmbH, Germany). The populations used in our study were from a later generation of the line that was studied previously [4, 17–19], and comprised 1262 females from three hatches, which were the offspring of 48 sires and 330 dams. Between 17 and 18 weeks of age, hens were housed in cages prior to commencement of egg laying, with a controlled regime of 16 h of light per day. Two eggs per hen were collected between 30 and 32 weeks of age (referred to as 31 weeks throughout this paper) for cuticle deposition determination, and again at 50 weeks of age.

#### Breeds 2A and 2B

Hens from breed 2 originated from two separate and closed pure lines (2A and 2B) of the White Leghorn (WL) breed, which lay white eggs and contribute to the breeding of commercial Lohmann Selected Layers (LSL; Lohmann Tierzucht GmbH, Germany). Breed 2B hens came from later generations of the same genetic line that was previously studied for bone traits [20], whereas breed 2A hens had not been studied previously. Breed 2A comprised 915 females, which were the offspring of 46 sires and 345 dams from two hatches and were housed in three huts at the same location. Breed 2B comprised 994 females, which were the offspring of 92 sires and 576 dams from a single hatch, and were housed in one hut at the same location. All hens were housed in individual cages from 17 to 18 weeks of age, with a regime of 16 h of light per day prior to commencement of egg laying. Two eggs per hen were collected for cuticle deposition determination, at 32 weeks of age for breed 2A and at 28 to 29 weeks of age for breed 2B (referred to as 29 weeks throughout this paper).

#### Breed 3

This population was a pure broiler breed that lays tinted eggs and contributes to the breeding of meat-type poultry (Aviagen, Scotland). The hens were from a single generation and comprised 1459 females from 13 flocks, which were the offspring of 152 sires and 518 dams. Hens were reared in pens with a single male and the source of each egg was ascertained from nest box recording. Two eggs per hen were collected at 39 weeks of age for cuticle deposition determination.

#### Breed 4

This population comprised hens from a single generation of White Rock (WR), which is a pure breed laying brown eggs and contributing to the female used to produce Lohmann Brown commercial layers (Lohmann Tierzucht GmbH, Germany). The population included 1374 females, which were the offspring of 67 sires and 521 dams and were housed in cages in one hut at the same location. Two eggs per bird were collected, between 60 and 63 weeks of age, for cuticle deposition determination (referred to as 62 weeks of age throughout this paper).

### Measurement of traits

#### Cuticle staining

Cuticle deposition was measured after staining with a combination of tartrazine and lissamine green [15]. For breeds 1, 2A, and 3, we used a commercial preparation (MST Cuticle Blue, M.S. Technologies Ltd, England) [4], whereas for breeds 2B and 4, we prepared the staining solution as described in [15]. A photograph that illustrates the different levels of cuticle staining is in Figure S2 (see Additional file 1: Figure S2). For eggs with the best cuticle, the staining is strong and regular over the whole surface, whereas for eggs with a poor cuticle, the staining is predominately very weak.

#### Measurement of stained cuticle

During the course of this project, the technology was progressively improved to accelerate data acquisition, but the basic principle remained the same throughout. The amount of light absorbed at 640 nm by the cuticle-bound tartrazine and lissamine green stain was used as the gold standard for cuticle deposition. Absorbance at 640 nm was measured using reflectance spectrometry on the intact egg prior to staining (Pre-stain Abs@640 nm) and after staining (Post-stain Abs@640 nm), and the difference before and after staining (ΔAbs @640 nm) was used to estimate cuticle deposition [4]. The Pre-stain Abs@640 nm is a measure of the depth of ‘brown-ness’ in the pigmentation of the eggshell, as the peak of protoporphyrin absorbance is around 644 nm [21], and this peak in absorbance is clearly demonstrated in a transmission scan of brown eggs [22].

For all breeds, two eggs per hen were used to estimate cuticle deposition but only one measurement per egg was recorded since within-egg variance was small [4]. The data from breed 2A showed that variation in the Pre-stain Abs@640 nm measurement was negligible (< 0.009) compared to that in Post-stain Abs@640 nm, thus no Pre-stain Abs@640 nm measurement was recorded for breed 2B and the post-stain measurement was used as the value of ΔAbs@640 nm. In other words for white eggs we assumed the Pre-stain Abs@640 nm was zero.

For breed 1, measurements were recorded with a USB4000-VIS–NIR spectrometer coupled to an ISP-REF integrating sphere and the data were collected using the Oceanview spectroscopy software (Ocean Optics, Oxford, England). For breeds 2A and 3, we used a custom-made stand-alone device, the EggometerV2, which is based on the same methodology as the spectrometer but has an improved speed of data acquisition and robustness. The EggometerV2 combined a tungsten halogen light source (Ocean Optics, Oxford, England), a custom-made optical-fiber reflectance probe (Ocean Optics, Oxford, England), and a fiber-coupled spectrometer (FLAME-S-UV–VIS-ES, Ocean Optics, Oxford, England) that was optimized for the visible spectrum. For breeds 2B and 4, we used an Ecutimeter 3 (Lomond Instruments, Kinross, Scotland), which is a next-generation prototype with custom software for rapid, automated data collection and reporting of parameters such as absorbance at a particular wavelength, in this case at 640 nm. In addition, among other refinements, the Ecutimeter 3 incorporates a silica window to protect the probe tip and allow easy cleaning. For all breeds, the instruments were calibrated using a WS-1 diffuse reflectance PTFE standard (Ocean optics, Oxford, England). Absorbance (A) is a unit-less measurement and was calculated as $A = - \log_{10} R$, where $R$ is the reflectance expressed relative to the WS-1 white standard.

#### Fluorescence and UV absorption measurement

As a possible alternative to staining and measurement at 640 nm, the intensity of tryptophan fluorescence was measured on eggs obtained from breed 1 (both ages), 2A, and 3, by using a custom-built fluorimeter. The fluorimeter employed an epifluorescence excitation/detection geometry, which uses the same objective lens to focus excitation light onto the sample and collect the emitted fluorescence. The excitation source was a 290-nm fiber-coupled LED (Ocean Optics, Oxford, England) and the spectrum of the emitted fluorescence (with emission maximum at 330 nm) was measured by a fiber-coupled spectrometer (FLAME-S-UV-VIS-ES, Ocean Optics, Oxford, England).

For one batch of 48 eggs from breed 1, we performed UV reflectance measurements to determine the absorbance at 290 nm of the cuticle and the underlying shell matrix. Measurements were made on unstained eggs, using the same optical system as in the Ecutimeter 3 (described above), but with the tungsten-halogen source replaced by the 290-nm LED. The absorbance at 290 nm was measured on the intact egg with respect to the WS-1 reflectance standard, and then on the same egg after removal of the cuticle. For both steps, three measurements were recorded in the area around the egg’s equator and averaged. The difference before and after removing the cuticle corresponds to the absorbance of the cuticle.

#### Color and shininess traits measured by Minolta colorimetry

L*a*b* scores were available for hens from breeds 1 and 2A. These traits were measured at Lohmann Tierzucht by using a Minolta colorimeter (Konica Minolta, Langenhagen, Germany) on a single egg that had been laid by the same hens as those contributing to this study, although the age of the hens at which eggs were laid differed, i.e. either 35 or 48 weeks of age. The colorimeter measured: (1) the luminance, L*, which ranges from dark to light; (2) the color on the green–red axis, a*, which ascends to ‘more red’; (3) the color on the blue-yellow axis, b*, which ascends to ‘more yellow’ [23]; (4) a combination L*a*b* score, which provides a numerical value for the perceived color that was made to have a positive sign for the desired breeding direction using the following equations, i.e. darker for brown eggs (100 − L* + a* + b*) and lighter for white eggs (L* + a* − 2b*); and the (5) ‘shininess’, i.e. the relative surface gloss or matt appearance of brown eggs, which derives from the amounts of light diffused or reflected back to the colorimeter [24]. Shininess was only available for breed 1 at 35 weeks of age. In addition, a subjectively scored dark brown spots (speckles) on the eggshell surface were measured on the same egg Minolta colorimetry, it is a subjective score ranging from 1 (many and big dark spots) to 9 (no spots).

#### Production traits

Traits related to production were available for breeds 1 and 2A and included: total egg production over the production period; egg weight, shape index, and mottling; body weight and feed intake at 30 weeks of age; and the breaking strength of eggs, measured at 35 and 48 weeks of age, using quasi-static compression at the poles. The methods used to make these measurements have been described previously [18, 25] and were analyzed in the same way as the other traits as described in the statistical methods below.

### Statistical methods

All analyses were carried out using mixed linear models fitted in R using the ASReml R-4 plug-in (VSN International, Hemel Hempstead, UK). Models were fitted separately within each breed. Multivariate models were used, for which a bivariate example is presented in the following. The model had the following terms:

$$\left[ {\begin{array}{*{20}c} {{\mathbf{y}}_{1} } \\ {{\mathbf{y}}_{2} } \\ \end{array} } \right] = \left[ {\begin{array}{*{20}c} {{\varvec{1}}\mu_{1} } \\ {{\varvec{1}}\mu_{2} } \\ \end{array} } \right] + \left[ {\begin{array}{*{20}c} {{\mathbf{X}}_{1} } & {\varvec{0}} \\ {\varvec{0}} & {{\mathbf{X}}_{2} } \\ \end{array} } \right]\left[ {\begin{array}{*{20}c} {{\varvec{\upbeta}}_{1} } \\ {{\varvec{\upbeta}}_{2} } \\ \end{array} } \right] + \left[ {\begin{array}{*{20}c} {{\mathbf{Z}}_{1} } & {\varvec{0}} \\ {\varvec{0}} & {{\mathbf{Z}}_{2} } \\ \end{array} } \right]\left[ {\begin{array}{*{20}c} {{\mathbf{u}}_{1} } \\ {{\mathbf{u}}_{2} } \\ \end{array} } \right] + \left[ {\begin{array}{*{20}c} {{\mathbf{e}}_{1} } \\ {{\mathbf{e}}_{2} } \\ \end{array} } \right],$$

where ${\mathbf{y}}_{1}$ and ${\mathbf{y}}_{2}$ are column vectors of two traits (where _1_ and _2_ are taken from the traits described in the above sections ‘Measurement of stained cuticle’, ‘Fluorescence and UV absorption measurement’, ‘Color shininess traits measured by Minolta colorimetry’ and ‘Production traits’); $\mu_{1}$ and $\mu_{2}$ are their respective means with **1** a column vector of 1’s; ${\varvec{\upbeta}}_{1}$ and ${\varvec{\upbeta}}_{2}$ are additional fixed effects for hatches or flocks or tiers according to the design for each breed, with ${\mathbf{X}}_{1}$ and ${\mathbf{X}}_{2}$ their respective design matrices; ${\mathbf{u}}_{1}$ and ${\mathbf{u}}_{2}$ are vectors of breeding values for the traits, with ${\mathbf{Z}}_{1}$ and ${\mathbf{Z}}_{2}$ their respective design matrices; and ${\mathbf{e}}_{1}$ and ${\mathbf{e}}_{2}$ are the residual errors. The fixed effects in addition to the mean were 2 degrees of freedom (df) per trait for breed 1, 1 df per trait for breed 2A, 2 df per trait for breed 4, and 12 df for breed 3. There were no additional fixed effects for breed 2B. The breeding values $({\mathbf{u}}_{1}^{\text{T}} , {\mathbf{u}}_{2}^{\text{T}} )^{\text{T}}$ were assumed distributed as ${\text{MVN}}\left( {0, {\mathbf{A}} \otimes {\mathbf{U}}} \right)$, where ${\mathbf{A}}$ is the numerator relationship matrix generated from the pedigree (the depth of the pedigree for Breed 1, 2A, 2B and 4 was 5 generations; breed 3 it was 7 generations), and ${\mathbf{U}}$ is a $2 \times 2$ matrix of genetic (co)variances; for $t$ traits, ${\mathbf{U}}$ was $t \times t$. For elements of ${\mathbf{U}}$, the genetic variance for trait $i$ is denoted $\upsigma_{{{\text{A}},i}}^{2}$ and the covariance between traits $i$ and $j$ is ${\text{r}}_{{{\text{A}},ij}}\upsigma_{{{\text{A}},i}}\upsigma_{{{\text{A}},j}}$, where ${\text{r}}_{{{\text{A}},ij}}$ is their additive genetic correlation. The residuals $({\mathbf{e}}_{1}^{\text{T}} , {\mathbf{e}}_{2}^{\text{T}} )^{\text{T}}$ were assumed distributed as ${\text{MVN}}\left( {0, {\mathbf{I}} \otimes {\mathbf{V}}} \right)$, where ${\mathbf{I}}$ is the identity matrix and ${\mathbf{V}}$ is a $2 \times 2$ matrix (or $t \times t$ for $t$ traits). For elements of ${\mathbf{V}}$, the residual variance for trait $i$ is denoted $\upsigma_{{{\text{E}},i}}^{2}$ and the covariance between traits $i$ and $j$ is ${\text{r}}_{{{\text{E}},ij}}\upsigma_{{{\text{E}},i}}\upsigma_{{{\text{E}},j}}$, where ${\text{r}}_{{{\text{E}},ij}}$ is their environmental correlation. For trait $i,$ the phenotypic variance was calculated as $\upsigma_{{{\text{P}},i}}^{2} =\upsigma_{{{\text{A}},i}}^{2} +\upsigma_{{{\text{E}},i}}^{2}$ and the heritability was calculated as $h_{i}^{2} =\upsigma_{{{\text{A}},i}}^{2} /\upsigma_{{{\text{P}},i}}^{2}$. Preliminary analyses showed no evidence (P > 0.05) of maternal effects associated with the dams of the hens that laid the eggs.

Hypothesis testing was conducted using maximum likelihood ratio tests for H_0_: $h_{i}^{2} = 0$ against the alternative H_1_: $h_{i}^{2} > 0$ and, where appropriate, for H_0_: ${\text{r}}_{{{\text{A}},ij}} = 1$ against the alternative H_1_: ${\text{r}}_{{{\text{A}},ij}} < 1$. For these tests, the critical value of $\chi_{1}^{2}$ was adjusted following Self and Laing [26] since the null hypotheses lie on the boundaries. When ${\text{r}}_{{{\text{A}},ij}}$ was close to 1 (or bound by ASReml to 1), the 95% support interval for ${\text{r}}_{{{\text{A}},ij}}$ was calculated as the set of values of ${\text{r}}_{{{\text{A}},ij}}$ for which the drop in 2 $\times$ log-likelihood from its maximum value was less than the 95%-ile of $\chi_{1}^{2}$.

Our approach for analyzing the many traits that were measured across several breeds was to assess the relevant genetic and environmental parameters in three steps: (1) estimate the parameters for the Pre-stain Abs@640 nm, Post-stain Abs@640 nm, and ΔAbs@640 nm for each breed; since these three traits are linearly dependent, the results were obtained by fitting a bivariate model for two traits and deriving the parameters involving the third trait algebraically within ASReml; (2) test the hypothesis that tryptophan fluorescence could replace staining measures by estimating the parameters that relate fluorescence at 330 nm to the ‘gold standard’ ΔAbs@640 nm and the potential confounder Pre-stain Abs@640 nm; these parameters were obtained from a tri-variate model; and finally, (3), explore the use of Minolta colorimetry by estimating correlations of traits from the colorimeter with ΔAbs@640 nm and Pre-stain Abs@640 nm using a series of tri-variate and quadri-variate analyses. Furthermore, the data from a flock of White Leghorn hens at the Roslin Institute were used to obtain a phenotypic predictor of ΔAbs@640 nm based on L*, a* and b* using multiple linear regression which is described in detail in Additional file 2. The predictor was used as a trait (${\text{r}}_{640}$) in analyses for heritability and genetic correlation.

Meta-analyses [27] were used in steps (1) and (2) to summarize the variation in the parameter estimates obtained from the five breeds. To simplify the presentation of the results from step (3) for breed 1 (RIR), correlations between traits were estimated using the simple average of the values measured at the two ages, see the Results section and (Additional file 3: Tables S1 and S2), and provide evidence that this latter approach was reasonable because the genetic correlation was not distinguishable from 1.

## Results

### Summary statistics for cuticle measurements at 640 nm

Summary statistics related to cuticle measurement for each breed are in Table 1 and graphs of the distribution of Pre-stain Abs@640 nm and ΔAbs@640 nm are in Figure S3 (see Additional file 1: Figure S3). Variation in Pre-stain Abs@640 nm was very small for breed 2A, with the whiteness of the egg being ‘whiter’ than the standard used. For breed 1, phenotypic standard deviations for measurements at the two ages recorded were very similar.

**Table 1:** Summary statistics of 640 nm absorbance spectrophotometry measurements for determining shell color and cuticle deposition for all breeds and ages

	Age (weeks)	n	Mean	Standard deviation
*Breed 1, RIR*	31			
Pre-stain Abs@640 nm		1262	0.178	0.044
Post-stain Abs@640 nm		1262	0.421	0.091
ΔAbs@640 nm		1262	0.243	0.071
*Breed 1, RIR*	50			
Pre-stain Abs@640 nm		1262	0.208	0.039
Post-stain Abs@640 nm		1261	0.447	0.096
ΔAbs@640 nm		1261	0.239	0.080
*Breed 2A, WL*	32			
Pre-stain Abs@640 nm		914	-0.053	0.011
Post-stain Abs@640 nm		915	0.211	0.116
ΔAbs@640 nm		914	0.264	0.112
*Breed 2B, WL*	29			
Post-stain Abs@640 nm		994	0.511	0.109
*Breed 3, broiler*	39			
Pre-stain Abs@640 nm		1448	0.039	0.028
Post-stain Abs@640 nm		1444	0.286	0.100
ΔAbs@640 nm		1444	0.247	0.094
*Breed 4, WR*	62			
Pre-stain Abs@640 nm		1373	0.359	0.045
Post-stain Abs@640 nm		1368	0.647	0.072
ΔAbs@640 nm		1368	0.288	0.063

Note that comparison of mean values between breeds or ages is not valid because the measurements were made at different times and ages, and with different instruments and stain batches

The breeds are Rhode Island Red (breed 1, RIR), two groups of White Leghorn (WL, breeds 2A and 2B), White Rock (breed 4, WR) and a broiler (breed 3). Absorbance ($A$) is a unit-less measurement and was calculated according to the formula $A = - \log_{10} R$ where $R$ is the fraction of light reflected. Differences in ‘${\text{n}}$’ are due to broken or missing eggs for some hens

#### Estimates of heritability of shell color and cuticle deposition measurements at 640 nm

Evidence of genetic variation, i.e. $h_{i}^{2} > 0$, was found for all 640-nm traits and for all breeds (see Table 2). Estimates of heritability for Pre-stain Abs@640 nm traits differed between breeds (P < 0.01), with breeds 1 (31 weeks) and 2A exhibiting much lower estimates than the other breeds, which had estimates in excess of 0.5. These differences were not associated with either egg color or the instrument used. Although the value of $h^{2}$ is population-specific, the meta-analysis detected no statistical evidence of heterogeneity among breeds (P > 0.05) in the $h^{2}$ estimates for Post-stain Abs@640 nm and ΔAbs@640 nm. For breed 1, estimates of $h^{2}$ for ΔAbs@640 nm at the two ages were similar, but estimates differed more for Pre-stain Abs@640 nm. Summary estimates of $h^{2}$ from the meta-analysis across breeds were equal to 0.43 (SE 0.04) for Pre-stain Abs@640 nm and 0.38 (SE 0.04) for ΔAbs@640 nm. Among breeds, the highest estimate of $h^{2}$ for ΔAbs@640 nm was from breed 2B (0.53, SE 0.10; WL at 29 weeks) and the lowest from breed 4 (0.26, SE 0.06; WR at 62 weeks). There was no evidence of maternal effects from the dam of the hen laying the egg on ΔAbs@640 nm in any of the breeds studied.

**Table 2:** Estimates of heritability of shell color and cuticle deposition measurement at 640 nm by breed and age

	Age (weeks)	Pre-stain Abs@640 nm	Post-stain Abs@640 nm	ΔAbs@640 nm
Breed 1 (RIR)	31	0.30 (0.07)	0.33 (0.07)	0.41 (0.07)
Breed 1 (RIR)	50	0.58 (0.08)	0.46 (0.07)	0.46 (0.07)
Breed 2A (WL)	32	0.22 (0.07)	0.33 (0.08)	0.34 (0.08)
Breed 2B (WL)	29	–	0.53 (0.10)	0.53 (0.10)
Breed 3 (broiler)	39	0.56 (0.07)	0.41 (0.07)	0.37 (0.07)
Breed 4 (WR)	62	0.50 (0.07)	0.27 (0.06)	0.26 (0.06)
Meta-analysis^a^	–	0.43* (0.07)	0.38 (0.04)	0.38 (0.04)

Standard errors are in parentheses

^a^The values for the meta-analysis provide a summary value following Der Simonian and Laird [26] with a * indicating evidence of heterogeneity between groups

#### Genetic and environmental correlations of shell color and cuticle deposition traits at 640 nm between ages

Estimates of genetic and environmental correlations for measurement of a trait over the period-of-lay for the same bird were based on repeated measurements at 31 and 50 weeks in breed 1 (RIR) and are in Table 3. Estimates of the genetic correlation between ages for Pre-stain Abs@640 nm, Post-stain Abs@640 nm and ΔAbs@640 nm were all higher than 0.95 and the lower bounds of their 95% support intervals were all higher than 0.80 (0.905 for ΔAbs@640 nm). Estimates of genetic correlations between measurements by age were much higher than estimates of environmental correlations, which ranged from 0.58 (SE 0.04) for Pre-stain Abs@640 nm to 0.16 (SE 0.07) for ΔAbs@640 nm. Estimates of phenotypic correlations were intermediate between the estimates of genetic and environmental correlations, since they are a weighted average of the latter. The estimate of the phenotypic correlation for Post-stain Abs@640 nm was intermediate to those of the other two traits.

**Table 3:** Estimates of genetic, environmental, and phenotypic correlations of shell color and cuticle deposition measurements at 640 nm taken at 31 and 50 weeks of age

	Pre-stain Abs@640 nm	Post-stain Abs@640 nm	ΔAbs@640 nm
*Genetic correlation*			
Estimate (SE)	0.95 (0.04)	0.98 (0.03)	1^a^ (NA)
95% support interval	(0.843, 1]	(0.890, 1]	(0.905, 1]
*Environmental correlation*			
Estimate (SE)	0.58 (0.04)	0.36 (0.06)	0.16 (0.07)
*Phenotypic correlation*			
Estimate (SE)	0.70 (0.02)	0.60 (0.02)	0.51 (0.02)

The 95% support interval was obtained from the likelihood profile

Standard errors are in parentheses

^a^The estimate was bound in the analysis and the calculated SE is unavailable

#### Genetic and environmental correlations between cuticle deposition traits measured at 640 nm

Genetic correlations between traits are more complex than correlations for a given trait between ages, as shown by the differences observed between breeds (see Table 4). Among these, only the correlations between Pre-stain and Post-stain Abs@640 nm showed no evidence of differences between breeds, with all the estimates being positive and statistically different from 0 (P < 0.05), and with a pooled estimate of 0.55 (SE 0.05). This indicates that eggshell color explains ~ 0.3 (0.55^2^) of the genetic variation in Post-stain Abs@640 nm, irrespective of color; however the standard errors of the individual breed estimates were substantial (~ 0.10), and this sampling error could mask important differences.

**Table 4:** Estimates of genetic (${\text{r}}_{\text{A}}$), environmental (${\text{r}}_{\text{E}}$) and phenotypic (${\text{r}}_{\text{P}}$) correlations between shell color and cuticle deposition traits at 640 nm, and the corresponding regression coefficients (${\text{b}}_{\text{A}}$, ${\text{b}}_{\text{E}}$, ${\text{b}}_{\text{P}}$)

	Breed 1		Breed 2A	Breed 3	Breed 4	Meta-analysis
Egg color	Brown		White	Tinted	Brown	
Age (weeks)	31	50	32	39	62	
*Pre*-*stain Abs@640* *nm and Post*-*stain Abs@640* *nm*						
${\text{r}}_{\text{A}}$	0.50 (0.12)	0.58 (0.09)	0.48 (0.18)	0.57 (0.09)	0.58 (0.11)	0.55 (0.05)
${\text{r}}_{\text{E}}$	0.71 (0.03)	0.59 (0.06)	0.28 (0.06)	0.33 (0.07)	0.49 (0.05)	0.48*(0.09)
${\text{r}}_{\text{P}}$	0.64 (0.02)	0.58 (0.02)	0.33 (0.03)	0.44 (0.03)	0.51 (0.02)	0.50*(0.05)
${\text{b}}_{\text{A}}$	1.04 (0.29)	1.22 (0.23)	6.07 (2.59)	1.92 (0.37)	0.68 (0.15)	1.21*(0.27)
${\text{b}}_{\text{E}}$	1.41 (0.10)	1.57 (0.21)	2.61 (0.62)	1.56 (0.35)	0.94 (0.12)	1.42*(0.18)
${\text{b}}_{\text{P}}$	1.30 (0.05)	1.37 (0.07)	3.38 (0.35)	1.77 (0.12)	0.81 (0.04)	1.58*(0.20)
*Pre*-*stain Abs@640* *nm and* ΔAbs@*640* *nm*						
${\text{r}}_{\text{A}}$	0.02 (0.16)	0.13 (0.13)	0.42 (0.19)	0.31 (0.12)	− 0.32 (0.14)	0.11*(0.13)
${\text{r}}_{\text{E}}$	0.28 (0.06)	0.26 (0.09)	0.18 (0.07)	0.12 (0.08)	− 0.04 (0.07)	0.16*(0.06)
${\text{r}}_{\text{P}}$	0.19 (0.03)	0.19 (0.04)	0.24 (0.03)	0.21 (0.03)	− 0.14 (0.03)	0.14*(0.07)
*Post*-*stain Abs@640* *nm and ΔAbs@640* *nm*						
${\text{r}}_{\text{A}}$	0.88 (0.04)	0.88 (0.03)	0.99 (0.01)	0.96 (0.01)	0.58 (0.11)	0.92*(0.03)
${\text{r}}_{\text{E}}$	0.88 (0.02)	0.93 (0.01)	0.99 (0.01)	0.97 (0.01)	0.85 (0.02)	0.93*(0.02)
${\text{r}}_{\text{P}}$	0.87 (0.01)	0.91 (0.01)	0.99 (0.01)	0.97 (0.01)	0.78 (0.01)	0.90*(0.04)

Standard errors are in parentheses

*indicating evidence of heterogeneity between groups

The trait that is associated with functional effects that arise from variation in cuticle deposition is ΔAbs@640 nm. For this trait, the picture was clearest for populations that lay white or tinted eggs, i.e. breeds 2 (WL) and 3 (the broiler line), respectively, for which the relative variation in Pre-stain Abs@640 nm is sufficiently small such that all correlation estimates with Post-stain Abs@640 nm were higher than 0.95 (Table 4). Because the benefit from measuring Pre-stain Abs@640 nm was very limited, we did not measure it in breed 2B. For the brown-egg breeds (breeds 1 and 4), the picture was less clear, particularly for breed 4. The gold standard ΔAbs@640 nm is not independent of color (as measured by Pre-Stain Abs@640 nm), for the different sources of variation, unless the corresponding regression coefficient ($b$ in Table 4) of Post-stain on Pre-stain Abs@640 nm estimates are equal to 1 (as ΔAbs@640 nm = Post-stain Abs@640 nm − 1 × Pre-stain Abs@640 nm). The evidence in Table 4 shows that, for Breed 4, the use of $b$ = 1 is insufficient to remove the genetic association of ΔAbs@640 nm with color, resulting in a negative genetic correlation. Estimates of the genetic correlations between Pre-stain Abs@640 nm and ΔAbs@640 nm were statistically significantly different from 0 for breeds 3 and 4 (P < 0.05), suggestive for breed 2A (P < 0.1), but was not significant for breed 1 at either age (P > 0.1).

#### Measurement of tryptophan fluorescence at 330 nm without staining

The intensity of tryptophan fluorescence measured at 330 nm decreased between the two ages examined in breed 1 (${\text{n}}$ = 1262, mean ± SD = 3875 ± 1088 and ${\text{n}}$ = 1230, 1052± 241 at 31 and 50 weeks, respectively). There was also a large numerical difference between the values obtained for breed 1 (brown eggs) at both ages and breed 2A (white eggs) (${\text{n}}$ = 934, 13,478 ± 1272) at 29 weeks of age.

Measurement of tryptophan fluorescence at 330 nm was based on the hypothesis that this may avoid the need for staining, and that strong associations would exist between tryptophan fluorescence and ΔAbs@640 nm, particularly at the genetic level. The estimate of heritability of tryptophan fluorescence was quite high (Table 5), with a combined estimate from the meta-analysis of 0.48 (SE 0.05), with no statistical evidence of differences between the four estimates from the 3 breeds. Breed 1 had two measurements available, at 31 and 50 weeks, and the genetic correlation between them was high (0.98 SE 0.01), with an environmental correlation of 0.51 (SE 0.06) and a phenotypic correlation of 0.75 (SE 0.01). The 95% support interval for the estimate of genetic correlation was (0.93, 1] (Table 5).

**Table 5:** Estimates of heritability of tryptophan fluorescence intensity at 330 nm^a^ in unstained eggs and of genetic and environmental correlations with shell color and cuticle deposition traits measured at 640 nm

	Breed 1		Breed 2A	Breed 3
Egg color	Brown		White	Tinted
Age^b^ (weeks)	31	50	32	39
Heritability	0.44 (0.08)	0.58 (0.08)	0.36 (0.08)	0.53 (0.07)
*Correlation with Pre*-*stain Abs@640* *nm*				
Genetic	− 0.81 (0.06)	− 0.76 (0.05)	− 0.08 (0.22)	− 0.85 (0.04)
Environmental	− 0.53 (0.05)	− 0.63 (0.06)	− 0.06 (0.07)	− 0.39 (0.08)
Phenotypic	− 0.63 (0.02)	− 0.71 (0.02)	− 0.07 (0.04)	− 0.64 (0.02)
*Correlation with ΔAbs@640* *nm*				
Genetic	0.28 (0.14)	0.25 (0.13)	− 0.20 (0.18)	− 0.16 (0.13)
Environmental	− 0.21 (0.08)	− 0.11 (0.10)	− 0.20 (0.08)	0.00 (0.08)
Phenotypic	− 0.01 (0.03)	0.08 (0.04)	− 0.20 (0.01)	− 0.07 (0.03)

^a^Excitation wavelength was 290 nm and the emission was measured at 330 nm

^b^Age at which the eggs were laid, some estimates used eggs laid $\pm$ 1 week from the age indicated

However, across all breeds, there was little consistency in the relationship of tryptophan fluorescence with all traits measured by absorbance at 640 nm and estimates of genetic correlation all significantly differed from 1 and none differed significantly from 0. Estimates of the corresponding environmental and phenotypic correlations were also low. Tryptophan fluorescence was negatively associated to Pre-stain Abs@640 nm, both genetically and environmentally. These genetic and environmental associations were substantial in the brown (breed 1) and tinted (breed 3) egg laying breeds, but they were weak and not statistically different from 0 for the white-egg laying breed 2A. This means that in brown or tinted egg layers, higher tryptophan fluorescence was genetically correlated with lower pigmentation of the shell, whereas in white egg layers (breed 2A), fluorescence was not correlated with color (Table 5). However, the phenotypic correlation of tryptophan fluorescence and ΔAbs@640 nm or Pre-stain Abs@640 nm suggested that, in brown or tinted egg layers, tryptophan fluorescence explained a small fraction of the phenotypic variance in ΔAbs@640 nm, but a relatively large amount of the phenotypic variance in Pre-stain Abs@640 nm (Table 5). This was also true for the genetic correlation of tryptophan fluorescence and Pre-stain Abs@640 nm, where the values were large for the brown and tinted eggs. The genetic and phenotypic correlations were all negative between tryptophan fluorescence and Pre-stain Abs@640, indicating that the greater the amount of pigment on the shell, the lower the tryptophan fluorescence emitted.

#### UV absorption measurement before and after cuticle removal

On a subset of brown eggs (${\text{n}}$ = 48, breed 1), the absorbances at 290 nm measured before and after cuticle removal were equal to 1.94 (SE 0.06), and 1.74 (SE 0.07), respectively, with the difference in absorbance averaging 0.21 (SE 0.10). The difference in absorbance is attributable to the removal of the cuticle and represents how much light at 290 nm is absorbed by the cuticle.

#### Measurement of L*a*b* traits by Minolta colorimetry

Additional file 2 documents a prediction equation of absorbance at 640 nm from Minolta colorimetry traits L*a* b*, which has been denoted as ${\text{r}}_{640} .$ Table 6 shows the genetic correlations of L*, a*, b* and ${\text{r}}_{640}$ with Pre-stain Abs@640 nm and ΔAbs@640 nm on brown eggs from breed 1 and white eggs from breed 2A. Results for the heritability and the genetic correlations between the Minolta traits at the two ages are in Table S1 and S2, respectively (see Additional file 3: Tables S1 and S2). The colorimetry traits were measured on the same hens as the cuticle measurements but on eggs laid at different ages, so the phenotypic measurements would not necessarily be expected to be close to 1 (see Additional file 4,1 Table S3). However it would be expected that the genetic correlation of ${\text{r}}_{640}$ with Pre-stain Abs@640 nm for breed 1 would be close to 1 since the genetic correlation of Pre-stain Abs@640 nm across ages was 0.95 (see Table 3), and this is observed in Table 6. The genetic correlation of ${\text{r}}_{640}$ with ΔAbs@640 in Table 6 is also consistent with the genetic correlation of Pre-stain Abs@640 with ΔAbs@640 for breed 1 shown in Table 3. The results for Breed 2A are less clear since the variation in absorbance at 690 nm prior to staining in this breed was on a negligible scale (see Table 1).

**Table 6:** Estimates of genetic correlations of Pre-stain Abs@640 nm and ΔAbs@640 nm with traits recorded by Minolta colorimetry for the brown egg layer breed 1 and the white egg layer breed 2A

	Pre-stain Abs@640 nm		ΔAbs@640 nm	
	Breed 1	Breed 2A	Breed 1	Breed 2A
L*	− 0.929 (0.029)	0.202 (0.259)	− 0.176 (0.136)	0.265 (0.224)
a*	0.920 (0.037)	0.506 (0.194)	0.220 (0.141)	− 0.325 (0.179)
b*	0.145 (0.154)	0.577 (0.146)	0.155 (0.146)	0.353 (0.156)
L*a*b* index^1^	0.865 (0.050)	− 0.396 (0.175)	0.180 (0.137)	− 0.258 (0.163)
Brown Spot	− 0.261 (0.136)	−	− 0.084 (0.132)	−
Shininess	− 0.958 (0.046)	−	0.272 (0.143)	−
${\text{r}}_{640}$	0.935 (0.029)	− 0.539 (0.271)	0.130 (0.133)	− 0.369 (0.252)

Standard errors are in parentheses

The colorimeter traits and staining traits are not contemporaneous. For breed 1, all traits except shininess are the average of two measurements taken at different ages

^1^L*a*b* index differs between breeds and is defined in the methods

#### Cuticle and production traits

For all breeds, we found no evidence of non-zero genetic correlations of ΔAbs@640 nm with production traits, i.e. total egg production over the production period, egg weight between 26 and 48 weeks of age, shape index, body weight and feed intake at 30 weeks of age or breaking strength of eggs measured at 48 weeks of age using quasi-static compression at the poles (data not reported).

## Discussion

Our findings constitute an important step towards the implementation of selection for improved cuticle deposition on eggs. The expected benefit is to decrease the likelihood of vertical transmission of pathogens from parent to offspring by reducing the entry of microorganisms into eggs, as well as improving food safety of table eggs that are directed to human consumption. We have both replicated and extended our previous observations [4] on the genetic parameters of this trait and, thus, have greatly increased the confidence in this approach. As indicated by the lack of heterogeneity in the meta-analysis, estimates of the heritability of cuticle deposition were substantial for all breeds, which indicates that about 38% of the variation in the trait is explained by genetics, which is more than adequate for allowing genetic progress. Furthermore, it is likely that, with technical improvements in staining and measurement of the cuticle, the proportion of variation attributable to genetics will increase, since undoubtedly some of the non-genetic variation is attributable to the reliance of the measurement on the consistency of staining the eggs. Although the estimate of heritability for cuticle deposition was high for all breeds, differences in estimates of heritability between breeds should be considered with caution given the differences in the instrumentations used within this study, the dates of the measurements, and the hens’ environments and ages. Our attempts to identify non-genetic factors involved in the variation of the cuticle suggest that stressors [15] and fixed environmental factors, such as hatch or house, have significant effects in this and other studies [28].

For poultry breeders, one of the most important correlations is the genetic correlation between early and later performance, which if it is high, would favor a single early measurement of a trait. Our results show that, for the cuticle, a single measurement at an early stage would be effective. In breed 1, for which measurements were made at two ages, we found a genetic correlation of 1 between measurements, with a tight 95% support interval. There was also no difference in the estimates of heritability between the measurements at two ages; therefore, our studies provide no evidence of benefit in measuring cuticle deposition at one age or another. Increasing age is typically associated with a decline in egg quality, color being a good example [29], which was replicated in this study and in a related longitudinal study [3], but also occurs for many other quality traits [30]. However, for cuticle deposition, we have no support for a decline in the trait with age, both in this study or in related longitudinal studies [3].

### Minolta colorimetry

In our studies, we used staining of the egg and spectrophotometric absorbance at a single wavelength to measure the cuticle deposition on eggs. In the layer breeding industry, the Minolta colorimeter is widely used to provide a measurement of shell color using the L*a*b* color space system. Our estimates of the genetic and environmental correlations of using an independently-derived Minolta prediction of Abs@640, which we termed r_640_, with the single-wavelength Pre-stain Abs@640 nm absorbance measurement was close to 1, even when eggs measured by Minolta and Pre-stain@640 nm were laid several weeks apart in this study. This means that breeders can use Minolta colorimetry with confidence to assess differences in staining to estimate cuticle deposition, as already reported in the literature [9, 31, 32]. In addition, the near perfect genetic correlations across ages for egg shell color in breed 1 confirms the widely accepted perception among breeders that egg color can be measured early or late in a hens life with similar results for selection; however, evidence for this does not seem to be widely reported.

### Cuticle and shell color

The most debated relationship is probably that between cuticle deposition and pigment. The argument centers on how much of the pigment is located in the cuticle [14, 33]. If significant amounts of pigment were in the cuticle, it seems plausible that the amount of cuticle is associated to the amount of pigment. In brown eggs, our physiological studies suggest that pigment deposition and cuticle deposition are distinct processes [15]. This is also supported by the estimated genetic correlation between these traits that is not significantly different from 0 in our previous studies [4], and by our current findings that show heterogeneity in the genetic correlation between the traits among the breeds studied. Our estimates of genetic correlation were either low or statistically not different from 0 for breeds that lay brown or tinted eggs, negative for breed 4, and positive for the tinted egg laying broilers, breed 3. However, for the white egg layer, breed 2A, we did observe a positive genetic correlation. Although pigment is minimal in white egg layers, the color perception may be affected by cuticle deposition, which is plausible because of the significant positive genetic correlation of cuticle deposition with the Minolta b* value, which indicates ‘yellower’ on the blue-yellow axis. However, overall, the positive, albeit non-significant, genetic correlation between cuticle deposition and luminance, L*, and significant negative correlations with a* and the L*a*b index would suggest that selection for cuticle deposition would also favor the selection of hens laying lighter whiter eggs. Although the authors of a study on the characterization of shell pigment observed that porphyrins are tightly bound to the shell mineral, they did point out that porphyrins would have been soluble when secreted into the shell gland at the end of shell formation and before oviposition [34]. This would at least allow for temporal secretion of cuticle and porphyrins, with subsequent binding of the porphyrins to the shell matrix and, therefore, give a physiological basis for a possible competition between the amount of shell pigment and cuticle. Thus, it is prudent to keep in mind that there is heterogeneity in the relationship between color and cuticle, although none of the observed genetic associations would prevent poultry breeding programs from moving both of these traits in the desired direction, regardless of the color of the eggs of the population. The relatively high genetic correlation of shininess with both cuticle deposition and tryptophan fluorescence is suggestive that shininess may be dependent on an aspect of the proteins in or on the shell. Given that shininess is almost certain to result from changes in the microcrystalline texture of the shell surface [35], it is possible that the proteins of the cuticle, or of the outer shell, modify the surface structure to give a finer texture and therefore more specular (non-diffuse) reflectance of light.

A further consideration is the genetic associations of cuticle deposition with production and quality traits other than color. Our analysis, over a wide range of egg quality and production traits, leads to the conclusion that there was, however, no evidence of any adverse correlation that would prejudice the use of cuticle deposition as a trait for selection.

In this study, we also tested an alternative method of assessing cuticle deposition that does not rely on staining the egg. We hypothesized that fluorescence intensity at 330 nm of the amino acid tryptophan from unstained eggs would be proportional to the amount of cuticle on the egg, since the cuticle is mainly composed of protein [4, 36, 37]. Measurement of tryptophan fluorescence proved to be rapid and repeatable, with heritabilities higher than 0.36. However, unfortunately this measure did not correlate strongly, either genetically or phenotypically, with the existing measurement of cuticle deposition. In breed 1, there was a reasonable genetic correlation between tryptophan fluorescence and cuticle deposition, but there was no evidence for a phenotypic correlation. For these reasons, we cannot recommend the use of tryptophan fluorescence as an indicator of cuticle deposition.

### Cuticle and tryptophan fluorescence

The failure to find significant correlations between cuticle deposition and tryptophan fluorescence was unexpected since tryptophan concentration is greater in the cuticle than in the shell. Our hypothesis was further undermined by the interaction between tryptophan fluorescence and shell color, as discussed below. An obvious explanation is that we are measuring tryptophan fluorescence from the proteins within the shell rather than just the cuticle; however, we found that removal of the cuticle resulted in an increase in tryptophan fluorescence intensity. This clearly indicates the presence of tryptophan-containing protein in the sub-cuticle eggshell matrix. Further investigation revealed that removal of the cuticle caused a decrease in the absorbance at 290 nm, which is characteristic of tryptophan. This is consistent with a previous observation that removal of the cuticle resulted in a significant increase in measured reflected light at ultraviolet wavelengths down to 300 nm in the eggs of chicken and of other species [38, 39]. For a sample of eggs from breed 1, we found that the absorbance for the cuticle was around 0.2, compared with a value of around 1.7 for the sub-cuticle matrix, which means that the cuticle absorbs about 40% of the incident UV light intensity at 290 nm, whereas the sub-cuticle shell matrix absorbs about 98% of the incident light at the same wavelength after cuticle removal. If one considers that the thickness of the cuticle is about 1/50th of that of the underlying shell, the absorbance values suggest that the concentration of tryptophan is about 6 times more in the cuticle than in the sub-cuticle matrix. The increase in tryptophan fluorescence intensity observed when the cuticle is removed shows that absorption of 290-nm light by the cuticle gives rise to less fluorescence than does its absorption in the sub-cuticular matrix; i.e., the fluorescence quantum yield of tryptophan is lower in the cuticle than in the underlying matrix. This quenching of tryptophan fluorescence in the cuticle is likely due to a combination of energy transfer to protoporphyrin pigment and self-quenching due to the high concentration of protein [40]. Removal of the cuticle allows a higher intensity of excitation light to reach the more emissive shell matrix, resulting in an increase in tryptophan fluorescence intensity; moreover, any attenuation of the matrix emission by absorption in the cuticle will be removed along with the cuticle. Thus, it is evident that the intensity of tryptophan fluorescence measured for an intact egg is made up of contributions from both the cuticle and the sub-cuticle matrix, and is subject to the complex influence of variations in the structure and composition of both components. This means that neither absorbance at 290 nm nor the fluorescence were related to cuticle quantity.

Large differences in the measured tryptophan fluorescence emission were observed between breeds, which may be explained in part by differences in protoporphyrin deposition between breeds, with the tryptophan fluorescence intensity being inversely related to the breed’s protoporphyrin deposition on the egg, which will quench the tryptophan fluorescence. A negative genetic and phenotypic correlation of egg color with tryptophan fluorescence was only evident in the breeds laying colored eggs. This suggests that fluorescence and pigment deposition are genetically linked, most likely due to the physical quenching of tryptophan fluorescence by protoporphyrin (the principal pigment in the shell of brown eggs [41]). Quenching occurs as a result of Forster resonance energy transfer [40] from excited tryptophan molecules to protoporphyrin molecules. In effect, light energy absorbed by a tryptophan molecule is transferred to a nearby protoporphyrin molecule, resulting in the fluorescence of tryptophan being suppressed and in emission occurring instead from protoporphyrin, at much longer wavelengths, between 620 and 700 nm. The greater the concentration of protoporphyrin, the greater the probability of energy transfer (quenching), resulting in a negative correlation between tryptophan fluorescence intensity and egg color. The effect of the cuticle in suppressing UV reflectance and tryptophan fluorescence from the egg at around 330 nm might suggests a role of the cuticle in camouflage [38, 39] at this wavelength. After all, many birds, including chickens, are thought to perceive light at this wavelength [42].

### Potential application

Simplicity of measurement is important for implementation of a new trait in a breeding program. For white eggs (breed 2), we have shown that the very low background level of protoporphyrin absorption makes a pre-stain measurement unnecessary; cuticle deposition can be estimated simply from only a post-stain measurement. For brown eggs (e.g. RIR, breed 1), the situation is less simple and the difference between pre- and post-stain measurements must be calculated to obtain an accurate estimate of the amount of cuticle. For tinted broiler eggs (breed 3), we found that a single post-stain measurement had a large enough genetic correlation with cuticle deposition to obtain satisfactory results, particularly since, for broilers, correlated changes in color are of no economic value.

In view of the favorable effects on bacterial penetration previously observed [4] and since replicated [9, 30], we believe that selection for cuticle deposition will contribute to reducing the risk of vertical transmission of avian pathogens, and reduce the incidence of infected eggs in the incubation process. In wild birds, the pressure to maintain a good cuticle is likely strong, which, to some extent, is supported by the observation that cuticle deposition is enhanced in species that have dirty nests, in particular eggs of aquatic birds [7]. It is conceivable that the almost universal use of artificial incubation, including at the pedigree level in a breeding program, and the relatively clean bio-secure environment in which breeding hens are kept, have reduced natural selection pressure on the deposition of cuticle. Given that the environment of chickens in production may contain greater challenges than in pedigree farms, we think considerable benefit can be accrued by incorporating this measurement into selection programs of both egg- and meat-type chickens.

## Conclusions

We have demonstrated, across independent breeds, a moderate heritability of cuticle deposition, which is adequate for genetic progress to increase the deposition of the cuticle. We have developed a simple-to-use instrumentation or suggested deployment of existing technology to be used in combination with a straightforward staining protocol to quantify the cuticle for genetic estimation. The results suggest that one measurement during the life of a hen is adequate to estimate genetic merit for the trait. Furthermore, we found no genetic correlations that could cause a problem in selection programs, assuming continued selection for shell color traits takes place. Overall, the trait of cuticle deposition can form part of a strategy to reduce the incidence of transmission of microorganisms in poultry breeding and reduce disease in production animals.

## Additional files

**Additional file 1: Figure S1.** Scanning electron micrograph of a transverse section of an eggshell showing the constituent parts of the calcified shell and on the outer surface, the cuticle. Annotated electron microscopy image of the eggshell. **Figure S2.** Eggs from Breed 2B stained with Lissamine green and Tartrazine at 29 weeks of age showing different levels of cuticle deposition. Photograph of stained eggs selected from the top and tail of the distribution of all eggs examined. **Figure S3**. Distribution of ΔAbs @640 nm and Pre-stain Abs@640 nm representing cuticle deposition and egg color. Histograms of the distribution of ΔAbs @640 nm and Pre-stain Abs@640 nm for Breed 1 at two ages and Breed 2A laying brown and white eggs, respectively.

**Additional file 2**. Correlation of 640 nm absorbance and L*a*b* values from eggs from a Roslin White leghorn population [15, 43]. Description of the rationale why the Minolta colorimeter can be used to determine cuticle deposition and the derivation of the trait r_640_.

**Additional file 3: Table S1.** Age correlations for Minolta colorimetry traits on eggs from Rhode Island Red hens: Heritabilities and phenotypic variances for six traits measured by Minolta colorimetry at 38 and 48 weeks. Heritabilities and phenotypic variances for L*, a*, b*, L*a*b*, Brown Spot and r640. **Table S2.** Title: Age correlations for Minolta colorimetry traits on eggs from Rhode Island Red hens: Estimates of correlations between 35 and 48 weeks of age for genetic variance (rG), environmental variance (rE), and phenotypic variance (rP). Genetic correlations were consistently very high and did not differ from 1.

**Additional file 4: Table S3.** Residual correlations of Minolta colorimetry measures with Pre-Stain 640 nm and Δ640 nm. Residual correlations obtained from fitting the multivariate mixed linear models described in Methods to Minolta colorimetry data and data obtained to measure cuticle deposition.

## Data Availability

The datasets analyzed during the current study containing pedigree information are not publicly available due to commercial sensitivity but analyzed data is available from the corresponding author on reasonable request. The data has been archived with the following 10.7488/0cb81910-40ee-49e8-b421-0c08b41985a8.

## References

[1] Gilbert AB, Bell DJ, Freeman BM (1971). The egg: Its physical and chemical aspects. Physiology and biochemistry of the domestic fowl.

[2] Vadehra DV, Baker RC, Naylor HB (1970). Role of cuticle in spoilage of chicken eggs. J Food Sci.

[3] Bain MM, Zheng J, Zigler M, Whenham N, Quinlan-Pluck F, Jones AC (2019). Cuticle deposition improves the biosecurity of eggs through the laying cycle and can be measured on hatching eggs without compromising embryonic development. Poult Sci.

[4] Bain MM, McDade K, Burchmore R, Law A, Wilson PW, Schmutz M (2013). Enhancing the egg’s natural defence against bacterial penetration by increasing cuticle deposition. Anim Genet.

[5] Kusuda S, Iwasawa A, Doi O, Ohya Y, Yoshizaki N (2011). Diversity of the cuticle layer of avian eggshells. J Poult Sci.

[6] von Nathusius W (1893). Die entwicklung von schale und schalenhaut des hühnereies im oviduct. Z Wiss Zool Abt A.

[7] D’Alba L, Maia R, Hauber ME, Shawkey MD (2016). The evolution of eggshell cuticle in relation to nesting ecology. Proc Biol Sci..

[8] Brackenridge CJ (1960). Optimal staining conditions for the quantitative analysis of human serum protein fractions by cellulose acetate electrophoresis. Anal Chem.

[9] Chen X, Li X, Guo Y, Li W, Song J, Xu G (2019). Impact of cuticle quality and eggshell thickness on egg antibacterial efficiency. Poult Sci.

[10] Sparks NHC, Board RG (1984). Cuticle, shell porosity and water-uptake through hens eggshells. Br Poult Sci.

[11] Board RG, Halls NA (1973). Cuticle—barrier to liquid and particle penetration of shell of hen’s egg. Br Poult Sci.

[12] Stanley WA, Hofacre CL, Ferguson N, Smith JA, Ruano M (2003). Evaluating the use of ultraviolet light as a method for improving hatching egg selection. J Appl Poult Res.

[13] Sparks NHC, Burgess AD (1993). Effect of spray sanitizing on hatching egg cuticle efficacy and hatchability. Br Poult Sci.

[14] Lang MR, Wells JW (1987). A review of eggshell pigmentation. Worlds Poult Sci J.

[15] Wilson PW, Suther CS, Bain MM, Icken W, Jones A, Quinlan-Pluck F (2017). Understanding avian egg cuticle formation in the oviduct: a study of its origin and deposition. Biol Reprod.

[16] Kranis A, Gheyas AA, Boschiero C, Turner F, Yu L, Smith S (2013). Development of a high density 600 K SNP genotyping array for chicken. BMC Genomics..

[17] Dunn IC, Rodriguez-Navarro AB, McDade K, Schmutz M, Preisinger R, Waddington D (2012). Genetic variation in eggshell crystal size and orientation is large and these traits are correlated with shell thickness and are associated with eggshell matrix protein markers. Anim Genet.

[18] Dunn IC, Joseph NT, Bain M, Edmond A, Wilson PW, Milona P (2009). Polymorphisms in eggshell organic matrix genes are associated with eggshell quality measurements in pedigree Rhode Island Red hens. Anim Genet.

[19] Dunn IC, Bain M, Edmond A, Wilson PW, Joseph N, Solomon S (2005). Heritability and genetic correlation of measurements derived from acoustic resonance frequency analysis; a novel method of determining eggshell quality in domestic hens. Br Poult Sci.

[20] Dunn IC, Fleming RH, McCormack HA, Morrice D, Burt DW, Preisinger R (2007). A QTL for osteoporosis detected in an F-2 population derived from White Leghorn chicken lines divergently selected for bone index. Anim Genet.

[21] Oliver IT, Rawlinson WA (1955). The absorption spectra of porphyrin-A and derivatives. Biochem J.

[22] Mertens K, Vaesen I, Loffel J, Kemps B, Kamers B, Perianu C (2010). The transmission color value: a novel egg quality measure for recording shell color used for monitoring the stress and health status of a brown layer flock. Poult Sci.

[23] Mokrzycki WS, Tatol M (2011). Colour difference Delta E—a survey. Mach Graph Vis.

[24] Icken W, Cavero D, Schmutz M, Preisinger R. Shininess of eggs: a new selection tool to obtain the most attractive eggs. In: Proceedings of the XXI European symposium on the quality of poultry meat and the XV European symposium on the quality of eggs and egg products, World Poultry Science Association, 15–19 Sept 2013, Bergamo; 2013.

[25] Cordts C, Schmutz M, Preisinger R (2002). New alternatives for improving egg shell stability through breeding. Lohmann Inf.

[26] Self SG, Liang KY (1987). Asymptotic properties of maximum-likelihood estimators and likelihood ratio tests under nonstandard conditions. J Am Stat Assoc.

[27] DerSimonian R, Laird N (2015). Meta-analysis in clinical trials revisited. Contemp Clin Trials.

[28] Tuiskula-Haavisto M, Honkatukia M, Dunn IC, Bain MM, De Koning D, Preisinger R (2018). Validated QTL for egg shell quality in experimental and commercial laying hens. Anim Genet.

[29] Odabasi AZ, Miles RD, Balaban MO, Portier KM (2007). Changes in brown eggshell color as the hen ages. Poult Sci.

[30] Sirri F, Zampiga M, Berardinelli A, Meluzzi A (2018). Variability and interaction of some egg physical and eggshell quality attributes during the entire laying hen cycle. Poult Sci.

[31] Dominguez-Gasca N, Munoz A, Rodriguez-Navarro AB (2017). Quality assessment of chicken eggshell cuticle by infrared spectroscopy and staining techniques: a comparative study. Br Poult Sci.

[32] Leleu S, Messens W, De Reu K, De Preter S, Herman L, Heyndrickx M (2011). Effect of egg washing on the cuticle quality of brown and white table eggs. J Food Prot.

[33] Samiullah S, Roberts JR (2014). The eggshell cuticle of the laying hen. Worlds Poult Sci J.

[34] Gorchein A, Lim CK, Cassey P (2009). Extraction and analysis of colourful eggshell pigments using HPLC and HPLC/electrospray ionization tandem mass spectrometry. Biomed Chromatogr.

[35] Igic B, Fecheyr-Lippens D, Xiao M, Chan A, Hanley D, Brennan PRL (2015). A nanostructural basis for gloss of avian eggshells. J R Soc Interface..

[36] Wellman-Labadie O, Picman J, Hincke MT (2008). Antimicrobial activity of cuticle and outer eggshell protein extracts from three species of domestic birds. Br Poult Sci.

[37] Miksik I, Charvatova J, Eckhardt A, Deyl Z (2003). Insoluble eggshell matrix proteins - their peptide mapping and partial characterization by capillary electrophoresis and high-performance liquid chromatography. Electrophoresis.

[38] Fecheyr-Lippens DC, Igic B, D’Alba L, Hanley D, Verdes A, Holford M (2015). The cuticle modulates ultraviolet reflectance of avian eggshells. Biol Open.

[39] D’Alba L, Torres R, Waterhouse GIN, Eliason C, Hauber ME, Shawkey MD (2017). What does the eggshell cuticle do? A functional comparison of avian eggshell cuticles. Physiol Biochem Zool.

[40] Lakowicz JR (2006). Principles of fluorescence spectroscopy.

[41] Fischer H, Kögl F (1923). Zur Kenntnis der natürlichen Porphyrine (IV). Über das Coporphyrin, Hoppe-Seyler’s. Z Physiol Chem.

[42] Lewis PD, Gous RM (2009). Responses of poultry to ultraviolet radiation. Worlds Poult Sci J.

[43] Basheer A, Haley CS, Law A, Windsor D, Morrice D, Talbot R (2015). Genetic loci inherited from hens lacking maternal behaviour both inhibit and paradoxically promote this behaviour. Genet Sel Evol.

