# Supplementary material for: Genetic variation and potential for genetic improvement of cuticle deposition on chicken eggs
Source: Genet Sel Evol. 2019 Jun 4;51:25. doi: 10.1186/s12711-019-0467-5 (PMC6549311; doi:10.1186/s12711-019-0467-5)
Supplement: Supplementary file 1 — Additional file 1: Figure S1. Scanning electron micrograph of a transverse section of an eggshell showing the constituent parts of the calcified shell and on the outer surface, the cuticle. Annotated electron microscopy image of the eggshell. Figure S2. Eggs from Breed 2B stained with Lissamine green and Tartrazine at 29 weeks of age showing different levels of cuticle deposition. Photograph of stained eggs selected from the top and tail of the distribution of all eggs examined. Figure S3. Distribution of ΔAbs @640 nm and Pre-stain Abs@640 nm representing cuticle deposition and egg color. Histograms of the distribution of ΔAbs @640 nm and Pre-stain Abs@640 nm for Breed 1 at two ages and Breed 2A laying brown and white eggs, respectively. [file 12711_2019_467_MOESM1_ESM.pdf]

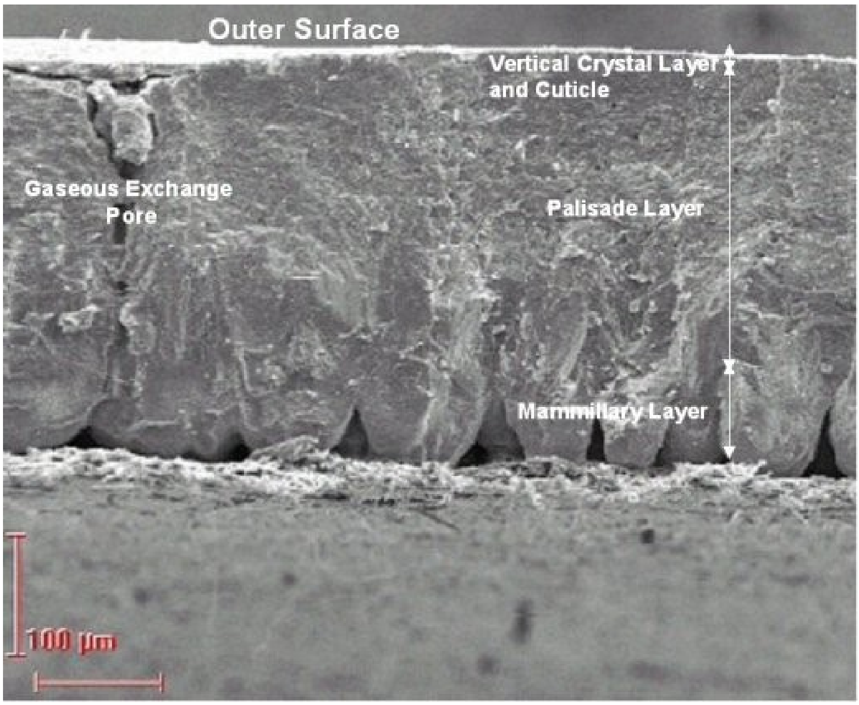

Supplementary figure 1. Scanning electron micrograph of a transverse section of an eggshell showing the constituent parts of the calcified shell and on the outer surface, the cuticle. The cuticle has formed a plug at the entrance to the gaseous exchange pore in this image and can be seen as a white layer (Image M. Bain, Glasgow University).

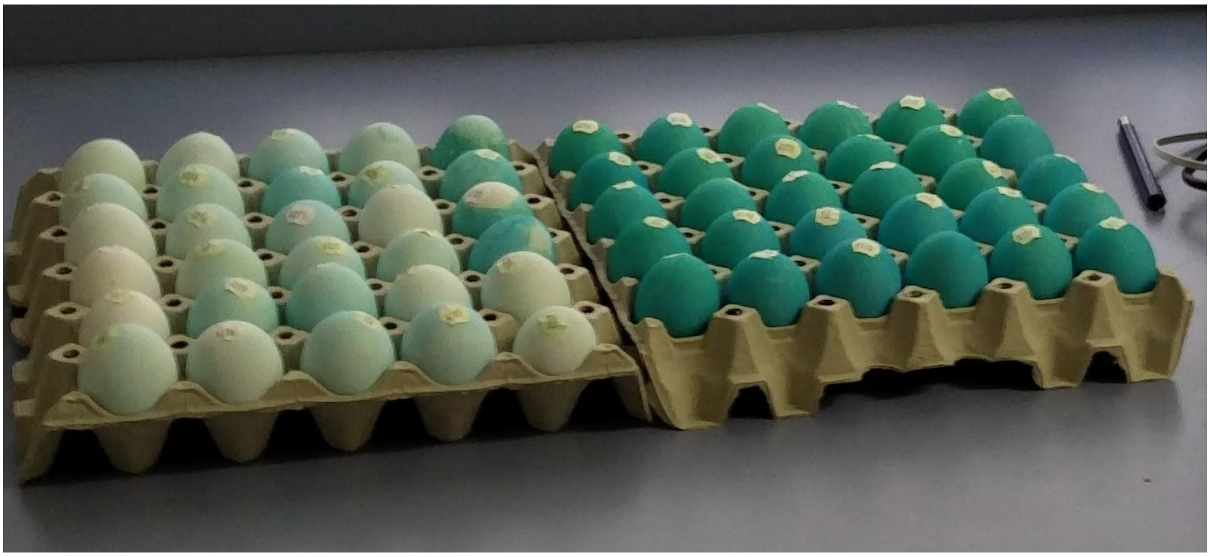

Supplementary figure 2. Eggs from Breed 2B stained with Lissamine green and Tartrazine at 29 weeks of age showing different levels of cuticle deposition. The eggs on the left egg tray are at the lower end of the cuticle deposition distribution whilst those on the right egg tray were at the higher end of the cuticle deposition distribution. Those on the right have a strong even staining. Those on the left have predominately very weak staining over the egg surface, but can be patchy.

RIR Breed 1

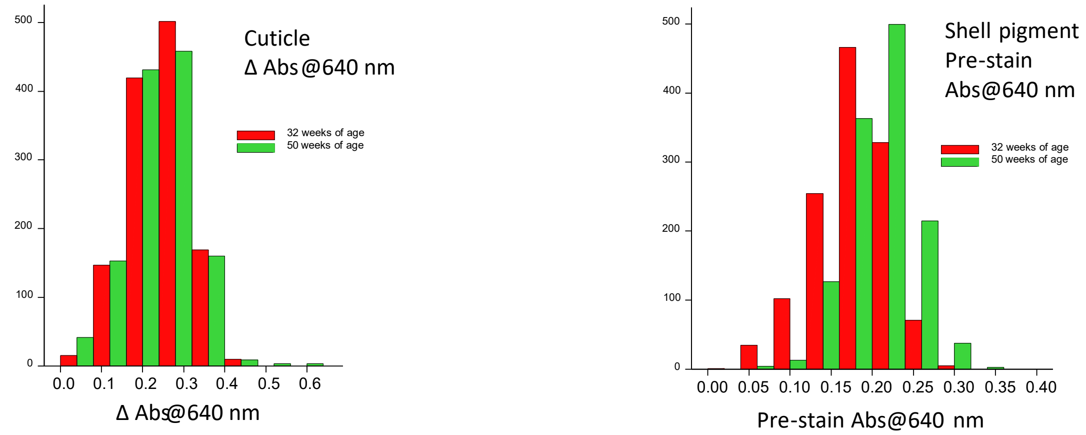

WL Breed 2A

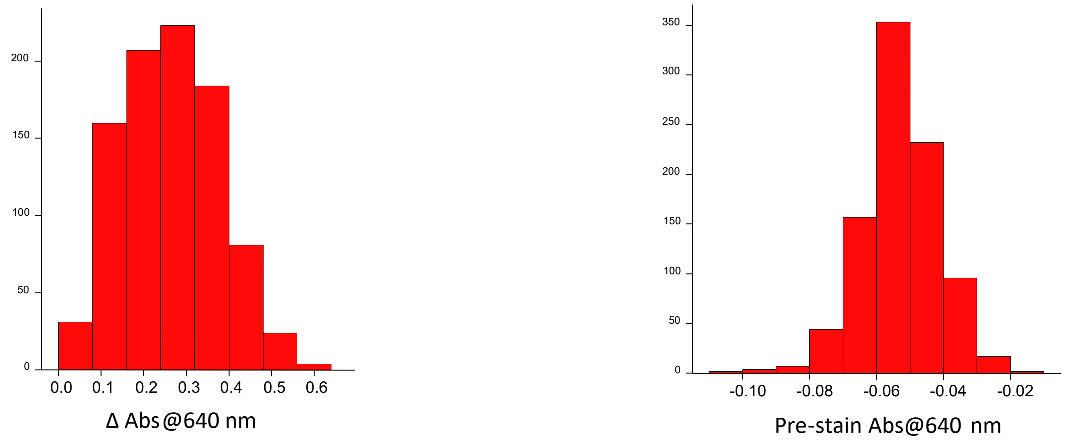

Supplementary Figure 3. The distribution of ΔAbs @640 nm and Pre-stain Abs@640 nm representing cuticle deposition and egg color. Data for breed 1 the RIR line which lays brown eggs at 2 ages, 32 and 50 weeks of age and Breed 2A a White Leghorn which lays white eggs at 32 weeks of age. There are similar distributions for cuticle deposition irrespective of age or Breed and what colour the egg it lays. Conversely the shell color distribution shows the expected differences between pre stain absorbance.
