# Supplementary material for: Genetic variation and potential for genetic improvement of cuticle deposition on chicken eggs
Source: Genet Sel Evol. 2019 Jun 4;51:25. doi: 10.1186/s12711-019-0467-5 (PMC6549311; doi:10.1186/s12711-019-0467-5)
Supplement: Supplementary file 2 — Additional file 2. Correlation of 640 nm absorbance and L*a*b* values from eggs from a Roslin White leghorn population [15, 43]. Description of the rationale why the Minolta colorimeter can be used to determine cuticle deposition and the derivation of the trait r640. [file 12711_2019_467_MOESM2_ESM.docx]

**Additional file 2**

**Relationship between 640 nm absorbance and L*a*b* values derived from eggs laid by hens from a Roslin White Leghorn population.**

*Materials and Methods.* A single egg at the peak of lay (33 weeks of age) from 117 eggs laid by White Leghorn hens and maintained at the institute [43] were used to compare the absorbance of stained eggs at 640 nm and the L*a*b* values. All measurements were recorded on a USB4000-VIS-NIR spectrometer coupled to an ISP-REF integrating sphere and data were collected using the Oceanview spectroscopy software (Ocean Optics, Oxford, England). Staining used tartrazine and lissamine green, as previously described [15].

*Results and Discussion.* ΔAbs@640 nm was regressed on changes in L*a*b*, i.e. ΔL*, Δa*, and Δb*. The regression was extremely accurate in predicting ΔAbs@640 nm with R^2^ > 0.995 on 113 d.f. and with coefficients –161 x10^-3^ (s.e. 4 x10^-3^), –76 x10^-3^ (s.e. 3 x10^-3^) and –27 x10^-3^ (s.e. 2 x10^-3^) for ΔL*, Δa*, and Δb* respectively.

The linear regression may serve as a predictor of absorbance at 640nm from L*a*b* measurements irrespective of the presence of staining. The population used here was distinct from all the populations used in the main study and the predictor, denoted r_640_ in the main paper, could be used more generally as a predictor of absorbance at 640 nm from Minolta L*a*b*. This was explored in Table 6.
