# Supplementary material for: Genetic variation and potential for genetic improvement of cuticle deposition on chicken eggs
Source: Genet Sel Evol. 2019 Jun 4;51:25. doi: 10.1186/s12711-019-0467-5 (PMC6549311; doi:10.1186/s12711-019-0467-5)
Supplement: Supplementary file 3 — Additional file 3: Table S1. Age correlations for Minolta colorimetry traits on eggs from Rhode Island Red hens: Heritabilities and phenotypic variances for six traits measured by Minolta colorimetry at 38 and 48 weeks. Heritabilities and phenotypic variances for L*, a*, b*, L*a*b*, Brown Spot and r640. Table S2. Title: Age correlations for Minolta colorimetry traits on eggs from Rhode Island Red hens: Estimates of correlations between 35 and 48 weeks of age for genetic variance (rG), environmental variance (rE), and phenotypic variance (rP). Genetic correlations were consistently very high and did not differ from 1. [file 12711_2019_467_MOESM3_ESM.docx]

**Additional File 3; Tables S1 and S2.**

**Age correlations for Minolta colorimetry traits on eggs from Rhode Island Red hens.**

As described in the text, two unstained eggs from Breed 1 (Rhode Island Red) were assessed using a Minolta colorimeter at 35 weeks of age, and a further two eggs from the same hens were assessed at 48 weeks of age. The measures obtained on both occasions were: L^*^, a^*^ and b^*^ (ref), L^*^a^*^b^*^, ‘Brown Spot’, and the linear predictor r_690_ = -0.0161 L^*^ - 0.0076 a^*^ - 0.0027 b^*^, where the latter follows the predictor obtained in Results. The Minolta index L^*^a^*^b^*^ is a combination of L^*^, a^*^ and b^*^.

The genetic and environmental correlations between ages were obtained using bivariate linear mixed models for each of the 6 traits. The models were implemented in ASReml 4 for R fitted in an R 3.5.1 environment. For further details see Materials & Methods.

Table S1 shows all the Minolta traits had moderate heritability at both ages. When tested, the differences in heritability between ages were not significantly different from each other (P>0.05), although there was a consisted trend for the heritability at the younger age to be the greater. There were also only small differences in the phenotypic variances at each age.

**Table S1.** Heritabilities and phenotypic variances for 6 traits measured by Minolta colorimetry at 38 and 48 weeks. Note the phenotypic variances for r_640_ have been scaled up by 10^4^.

|  | Heritability | | Phenotypic Variance | |
| --- | --- | --- | --- | --- |
| Age (weeks) | 35 | 48 | 35 | 48 |
| L^*^ | 0.32 (0.07) | 0.24 (0.06) | 2066 (96) | 1957 (87) |
| a^*^ | 0.25 (0.06) | 0.20 (0.06) | 516 (23) | 519 (23) |
| b^*^ | 0.22 (0.06) | 0.18 (0.06) | 530 (23) | 549 (24) |
| L^*^a^*^b^*^ | 0.28 (0.06) | 0.20 (0.06) | 64.1 (2.89) | 64.2 (2.89) |
| Brown Spot | 0.33 (0.07) | 0.31 (0.06) | 1.56.(0.07) | 1.36 (0.06) |
| r_640_ | 0.33 (0.07) | 0.25 (0.06) | 31.6 (1.5) | 28.5 (1.3) |

Table S2 shows that the genetic correlations were consistently very high and were all >0.97 and bound for three of the traits. There was no evidence these correlations differed from 1. The 95% support intervals all had lower bounds greater than 0.80. In contrast the environmental correlations were only moderately positive (~0.3), and consequently all the phenotypic correlations were intermediate ranging from 0.37 for b* to 0.50 for L*.

**Table S2.** Estimates of correlations between 35 and 48 weeks of age for genetic variance (r_G_), environmental variance (r_E_), and phenotypic variance (r_P_), and the 95% support interval for the estimate of r_G_.

|  | Support Interval |  | Correlations |  |
| --- | --- | --- | --- | --- |
|  | r_G_ | r_G_ | r_E_ | r_p_ |
| L* | (0.84, 1] | 0.98 (0.05) | 0.33 (0.05) | 0.50 (0.02) |
| a* | (0.86, 1] | 0.99 ( NA) | 0.33 (0.04) | 0.48 (0.02) |
| b* | (0.80, 1] | 0.98 (0.09) | 0.22 (0.05) | 0.37 (0.03) |
| L^*^a^*^b^*^ | (0.85, 1] | 0.99 ( NA) | 0.33 (0.04) | 0.48 (0.02) |
| Brown Spot | (0.88, 1] | 0.99 ( NA) | 0.31 (0.05) | 0.42 (0.03) |
| r_640_ | (0.83, 1] | 0.97 (0.05) | 0.31 (0.05) | 0.49 (0.02) |

Two conclusions can be made from these analyses. Firstly in Rhode Island Red the genetic correlation for variation in egg colour across the measured ages is very high. Secondly, a weighted average of the traits will maintain the genetic information and reduce the environmental variance, and, furthermore, equal weighting between the ages is approximately optimum due to the small scale of differences in heritability and phenotypic variance. The second conclusion simplified the approach to modelling the relationships of data from Minolta colorimetry with cuticle deposition data for in Rhode Island Red hens.
