# Supplementary material for: Genetic variation and potential for genetic improvement of cuticle deposition on chicken eggs
Source: Genet Sel Evol. 2019 Jun 4;51:25. doi: 10.1186/s12711-019-0467-5 (PMC6549311; doi:10.1186/s12711-019-0467-5)
Supplement: Supplementary file 4 — Additional file 4: Table S3. Residual correlations of Minolta colorimetry measures with Pre-Stain 640 nm and Δ640 nm. Residual correlations obtained from fitting the multivariate mixed linear models described in Methods to Minolta colorimetry data and data obtained to measure cuticle deposition. [file 12711_2019_467_MOESM4_ESM.docx]

**Additional file 4 Table S3 Residual correlations of Minolta colorimetry measures with Pre-Stain 640 nm and Δ640 nm.**

Table S3 shows the residual correlations obtained from fitting the multivariate mixed linear models described in the Materials and Methods to Minolta colorimetry data and data obtained to measure cuticle deposition. The colorimetry measures were obtained from a single egg laid by the same hens contributing to this study, although the eggs used differed since they were been laid at either 35 or 48 weeks of age. The colorimeter measured: (i) Luminance, L*, ascending from dark to light); (ii) color on the green-red axis, a*, ascending to ‘more red’; (iii) color on the blue-yellow axis, b*, ascending to ‘more yellow’; (iv) a combination L*a*b* score providing a numerical value for the perceived color; (v) ‘Brown Spot’, (vi) ‘Shininess’, and (vii) r_640_ a linear combination of L*, a*, and b* found to strongly predict changes in absorbance at 640 nm following staining for measuring cuticle deposition in a different population (see Results).

For Breed 2A (White Leghorn, which lays white eggs), the staining measures were taken at the single age of 32 weeks of age and are the average of two eggs, and the colorimetry measures are on a single egg taken from the same birds at 35 weeks of age. For breed 1 (Rhode Island Red, which lays brown eggs), the staining measures are the average of two ages, 31 and 50 weeks, and are the average of two eggs at each age taken from the same birds. The colorimetry measures are the average of single eggs taken at 35 and 48 weeks of age from the same birds, with the exception of Shininess, which was only measured at 35 weeks of age.

Table S3

|  | **Pre-Stain 640 nm** | | | **Δ640 nm** | |
| --- | --- | --- | --- | --- | --- |
|  | **Breed 1** | **Breed 2A** | **Breed 1** | | **Breed 2A** |
| L* | -0.632 (0.042) | -0.023 (0.053) | -0.229 (0.081) | | -0.134 (0.065) |
| a* | 0.589 (0.043) | -0.090 (0.072) | 0.184 (0.076) | | -0.076 (0.075) |
| b* | 0.305 (0.071) | 0.125 (0.077) | 0.027 (0.077) | | 0.071 (0.087) |
| L*a*b* | 0.585 (0.045) | -0.145 (0.080) | 0.206 (0.077) | | -0.153 (0.089) |
| Brown Spot | -0.005 (0.086) | - | 0.003 (0.092) | | - |
| Shininess | -0.365 (0.061) | - | -0.055 (0.080) | | - |
| r_640_ | 0.614 (0.043) | 0.016 (0.058) | 0.266 (0.081) | | 0.129 (0.063) |
